# Erythrocytes, a New Contributor to Age‐Associated Loss of Blood–Brain Barrier Integrity

**DOI:** 10.1002/advs.202101912

**Published:** 2021-08-16

**Authors:** Payam Amiri, Jonalyn DeCastro, Joshua Littig, Hsiang‐Wei Lu, Chao Liu, Irina Conboy, Kiana Aran

**Affiliations:** ^1^ Henry E. Riggs School of Applied Life Sciences Keck Graduate Institute Claremont CA 91711 USA; ^2^ Department of Bioengineering University of California, Berkeley Berkeley CA 94720 USA

**Keywords:** aging, blood–brain barriers, erythrocytes, microfluidics, organ‐on‐a‐chips

## Abstract

Blood exchanges between young and old partners demonstrate old blood has a detrimental effect on brain health of young animals. Previous studies primarily investigate soluble blood factors, such as transforming growth factor‐beta, on the brain and the blood–brain barrier (BBB). However, the role of blood cellular components, particularly erythrocytes, has not been defined. Erythrocyte morphology and rigidity change as mammals age, altering their transport within the capillary bed. This impacts downstream biological events, such as the release of reactive oxygen species and hemoglobin, potentially compromising the BBB. Here, a micro electrical BBB (µE‐BBB), with cocultured endothelial and astrocytic cells, and a built‐in trans‐endothelial electrical resistance (TEER) system is described to monitor the effect of capillary shear stress on erythrocytes derived from young and old mice and people and the subsequent effects of these cells on BBB integrity. This is monitored by the passage of fluorescein isothiocyanate‐dextran and real‐time profiling of TEER across the BBB after old and young erythrocyte exposure. Compared to young erythrocytes, old erythrocytes induce an increased permeability by 42% and diminished TEER by 2.9% of the µE‐BBB. These results suggest that changes in circulating erythrocytes are a biomarker of aging in the context of BBB integrity.

## Graphical abstract

The blood–brain barrier (BBB) is a vital structure that protects the brain from inhibitory molecules found in circulation. However, this barrier has been shown to become leaky with age. This study finds that erythrocytes derived from old mammals (mice and people) have deleterious effects on the integrity of the BBB, when compared to the young cells.

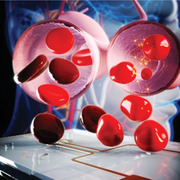

## 1 Introduction

Advancements in modern medicine have greatly extended human life expectancy. However, with the onset of age‐associated conditions, healthy aging is becoming an increasingly relevant pursuit. Recent studies have suggested that blood factors may play a critical role in the age‐related neurological deterioration of animals. For example, a single heterochronic blood exchange between a young and old mouse resulted in a drastic decline in hippocampal neurogenesis, agility, and learning in the young mouse, while showing no significant improvements on the brain health and function in the old animal.^[^ ^1^ ^]^ Other studies have found that certain circulating cytokines, such as interleukin‐6,^[^ ^2^ ^]^ transforming growth factor‐beta (TGF‐*β*),^[^ ^3^ ^]^ and tumor necrosis factor‐alpha^[^ ^4^ ^]^ are elevated with age, antagonizing tissue repair,^[^ ^5^ ^]^ inhibiting neurogenesis,^[^ ^3^ ^]^ and inducing chronic inflammation.^[^ ^6^ ^]^ The majority of these studies focus on soluble blood factors but neglect the role of cellular blood components and the phenotypic changes that occur in these cells upon aging. And little is known about the role of the age‐specific changes in circulating cells on brain health. Here we uncover the age‐specific effects of erythrocytes on the integrity of blood–brain barrier (BBB) with a mechanistic link to the age‐associated neurological decline, using an organ‐on‐a‐chip, micro electrical BBB model (µE‐BBB).

**Figure 1:**
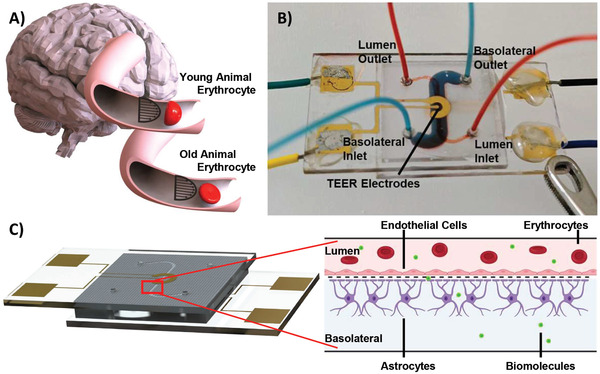
Schematic of the µE‐BBB device and erythrocyte deformability. A) The erythrocyte membrane deforms to a paraboloid shape to better accommodate the shear stress in the capillary beds. Here, the black arrows depict the flow profile of shear stress forces. Erythrocytes from old animals (bottom) cannot deform as readily as young animal erythrocytes (top), due to their greater rigidity, and retain their discus shape. B) The µE‐BBB consists of a lumen and basolateral chamber with integrated TEER electrodes. The chambers are separated by a porous polycarbonate (PC) membrane. C) The red square depicts a portion of the functional neurovascular growth area within µE‐BBB. Endothelial cells (pink) are grown within the lumen of the device. Astrocytes (purple) are grown within the basolateral compartment. Blood cells (red) and biomolecules (green) may pass through the lumen of the device, but only biomolecules that can cross the BBB would be present in the basolateral chamber. Figure 1c created with BioRender.com.

Erythrocytes, the most abundant cells in circulation, are vital gas transporters,^[^ ^7^ ^]^ and remain in the circulation for up to 120 days in humans and 60 days in mice. As animals age, erythrocytes undergo significant morphological changes. For example, old erythrocytes (even when recently generated) are more rigid and less deformable than the erythrocytes produced by a young mammal. Studies corroborating this phenomenon encompass multiple animal models and experimental set‐ups, including erythrocyte deformability and blood viscosity through centrifugal analysis and phase‐contrast microscopy and membrane fluidity studies of erythrocyte ghosts.^[^ ^8^, ^9^, ^10^, ^11^, ^12^, ^13^ ^]^ Previously published computational and real‐time microscopy research determined that when healthy young erythrocytes are subjected to a high shear stress environment, such as that of physiological capillaries, they deform from their native biconcave disk structure to a paraboloid.^[^ ^14^ ^]^ Due to increased rigidity, erythrocytes of old mammals deform less readily, which is predicted to increase the shear stress on these cells (**Figure** **1**a).^[^ ^12^ ^]^ This hypothesis is supported by our current work with the µE‐BBB organ‐on‐a‐chip (Figure 1).

Erythrocytes present the endothelial membrane‐bound nitric oxide synthase (eNOS) isoform. Interestingly, the high levels of shear stress found in capillary beds increase the activity of eNOS, subsequently increasing the production of the reactive oxygen species, and nitric oxide (NO)^[^ ^15^ ^]^ by erythrocytes, which was implicated in increased permeability of the BBB.^[^ ^16^, ^17^, ^18^, ^19^ ^]^ Supporting these findings, antioxidants restore BBB function^[^ ^20^, ^21^ ^]^ and denser and more rigid erythrocytes produce more NO.^[^ ^22^ ^]^ Additionally, erythrocytes in young mammals remain in circulation for a longer time, while in old animals erythrocytes have higher turnover rate.^[^ ^23^, ^24^, ^25^, ^26^ ^]^ The lysis of erythrocytes releases hemoglobin (Hb) into circulation and studies of cell‐free Hb found strong associations with oxidative stress and BBB damage.^[^ ^27^, ^28^ ^]^ Here we use µE‐BBB organ‐on‐a‐chip to explore mechanistic causal links between erythrocyte aging and the loss of BBB integrity, including NO production, hemolysis, and free Hb induced damage.

The BBB is characterized by tightly joined endothelial cells and supplemental basement membranes. It serves to isolate the central nervous system (CNS) from the circulating blood.^[^ ^29^ ^]^ Specifically, BBB presents high resistance to most blood‐derived products to prevent their diffusion into the CNS. This is crucial, as many circulating compounds are neurotoxic and/or neuro‐inflammatory to brain health, such as fibrinogen^[^ ^30^ ^]^ and TGF‐*β*.^[^ ^3^ ^]^ The tight junctions (TJs) of adjacent BBB endothelial cells play a pivotal role in maintaining its integrity.^[^ ^31^ ^]^ It has also been shown that advanced age is associated with the decline in the integrity of the BBB^[^ ^32^, ^33^ ^]^ but the underlying pathologies leading to loss of BBB integrity with age are yet to be understood. In addition, several neurological diseases are associated with a compromised BBB, such as Alzheimer's disease,^[^ ^33^, ^34^, ^35^ ^]^ Parkinson's disease,^[^ ^34^ ^]^ multiple sclerosis,^[^ ^36^ ^]^ and traumatic brain injury.^[^ ^37^ ^]^ These studies use a new technological platform for understanding the age‐specific multiparametric effects of erythrocytes on the integrity of the BBB, connecting these effects with the changes in cell morphology, biochemistry, and viability. This is significant in investigating the role of systemic milieu in preventing or promoting the passage of molecules to the brain and thus, the impact on neurological and age‐associated disease.

Studies utilizing both in vivo^[^ ^32^, ^38^ ^]^ and ex vivo^[^ ^39^, ^40^, ^41^, ^42^, ^43^, ^44^, ^45^ ^]^ models provided valuable information on the impact of various biomolecules on BBB, including in vivo work with calcium‐binding protein B,^[^ ^38^ ^]^ ex vivo studies with antineoplastic agent mitoxantrone,^[^ ^45^ ^]^ and research with other chemical compounds^[^ ^40^, ^42^, ^43^, ^44^, ^45^ ^]^ and endocrine factors.^[^ ^32^, ^38^ ^]^ A major challenge with these experimental systems is a lack of standardized quantification of barrier permeability, capillary induced shear stress, etc., parameters, which limits the potential for an accurate comparison of various factors to each other.^[^ ^39^, ^41^, ^43^, ^44^, ^46^ ^]^ To mitigate these problems, we developed an organ‐on‐a‐chip, µE‐BBB with built‐in trans‐endohelial electrical resistance (TEER) electrodes that provides real‐time feedback on BBB resistance. We show that TEER is inversely proportional to BBB permeability, thus providing an accurate and sensitive standard quantification of barrier permeability. Furthermore, the µE‐BBB can mimic the physiological flow‐induced shear stress that cells experience by taking advantage of specific flow properties incorporated into its channel design. The µE‐BBB is based on micro‐electro‐mechanical systems and is composed of a microlumen and a micro basolateral chamber, separated by a porous polycarbonate (PC) membrane (Figure 1b). Brain endothelial cells are cultured on the lumen side of the membrane while astrocytes are grown on the basolateral side of the membrane (Figure 1c). The integrated gold electrodes within the system allow for real‐time TEER monitoring across the BBB. The developed µE‐BBB was utilized to monitor the impact of old and young mouse and human erythrocytes on BBB integrity and passage of biomolecules.

Erythrocytes were isolated from the blood of old and young donors via Histopaque separation.^[^ ^47^ ^]^ The lumen channel of the device simulates a brain microcapillary and can induce low and physiological levels of shear stress. Based on Poiseuille's equation for laminar flow (Equation (1)), the shear stress within the lumen channel was calculated.^[^ ^48^ ^]^

(1) $$\tau \;=\left(4\eta \cdot Q\right)/\left(h^{2}\cdot w\right)$$

Isolated erythrocytes were passed through the lumen channel of the µE‐BBB at 40 µL min^−1^, simulating a physiological shear stress of 2 Pa.^[^ ^49^, ^50^ ^]^ Low shear stress conditions were induced by reducing the flow rate to 8 µL min^−1^, simulating 0.4 Pa of stress. This flow rate reflects the lower range of physiological shear stress that is both achievable within the device and in accordance with biological references of a low shear environment.^[^ ^51^, ^52^ ^]^ TEER measurements were obtained throughout the exposure to erythrocytes to shear stress to monitor the integrity of the BBB. Following this exposure, the passage of a fluorescent molecule typically unable to easily pass through a sufficiently tight BBB, fluorescein isothiocyanate‐dextran (10 kDa, FITC‐dextran), was monitored for its penetration from the lumen to the basolateral chamber, to further quantitatively assess the effects of the young and old erythrocytes on the µE‐BBB.

## 2 Experimental Section

### 2.1 µE‐BBB Design and Fabrication

Designed with Solidworks, the µE‐BBB was structured to feature a luminal channel and basolateral chamber, separated by a 0.4 µm pore PC membrane (WHA111107, Millipore). The lumen channel of the device simulated a brain microcapillary and could induce physiological levels of shear stress. Poiseuille's equation (Equation (1)),was used to calculate the shear stress within the lumen channel.^[^ ^48^ ^]^

The height (*h*) and width (*w*), of the lumen channel was 50 µm and 1 mm, respectively. The cell culture media's viscosity, *η*, used for the µE‐BBB was 1.2 mPa·s. A physiological shear stress of 2 Pa could be induced within the system when a flow rate, *Q*, of 40 µL min^−1^ was used in the system. Low shear stress could be induced by reducing the flow rate of the system to 8 µL min^−1^, achieving a shear stress of 0.4 Pa.

To properly simulate the in vivo BBB, the media must be under constant flow to mimic flow within the blood vessels. However, the brain's interstitial fluid on the basolateral side of the BBB was more static. Therefore, the basolateral chamber was designed to be static and larger, amounting to 1.5 mm in height and 3 mm in width, to mimic the BBB and allow for sample collection from the chamber.

One important aspect of the design of the µE‐BBB was the layout of the TEER electrodes. These electrodes were patterned by shadow mask sputtering, and the gold deposited to a thickness of 50 nm (K575XD, Quorum Technologies) onto precut pieces of PC stock (#2209, Grainger). The strategic layout of the electrodes in relation to the cells within the µE‐BBB directly affected the robust sensitivity of the system's TEER measurements. The µE‐BBB was designed to be compatible with the EVOM2 epithelial voltohmmeter (World Precision Instruments) utilizing an EVOM2 Electrical Adapter (World Precision Instruments). The EVOM2 required two apical and two basolateral electrodes. The electrode pairs were designed to align perpendicularly to the embedded PC membrane so that the uniform flow of ions between the electrodes minimized the noise of the system. The electrodes were designed with large contact pads to allow for wire soldering connections to the EVOM2. The 22‐gauge tinned copper wires were bonded onto the gold contact pads utilizing low temperature 52In/48Tn solder and the connections were reinforced with hot glue.

Another consideration of the system was rapid, reliable fabrication. To avoid the complexity of thin, multilayer fabrication utilizing traditional microfluidic materials such as polydimethylsiloxane, microfluidic tape was used instead. The microfluidic tape allowed for precise control of the thickness of the shear inducing lumen channel. The microfluidic tape had an added benefit of simplifying membrane excision for cellular imaging purposes. Exposure to isopropyl alcohol weakens the tape's adhesive, allowing for the user to extract the embedded PC membrane.

**Figure 2:**
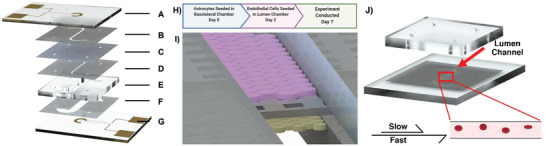
The µE‐BBB is comprised of A) a lumen electrode, B) a lumen channel, C) a PC membrane, D) a membrane‐basolateral adhesive layer, E) a basolateral layer, F) a basolateral electrode adhesive layer, and G) a basolateral electrode. H,I) The µE‐BBB is seeded with astrocytes (yellow) on day 0, and with endothelial cells (pink) on day 2. By day 7, the µE‐BBB is fully mature. J) To evaluate the behavior of erythrocytes in response to shear stress, a modified µE‐BBB was utilized that only contains the shear inducing lumen channel. Schematic shows that old and young erythrocytes experience either a slow flow rate (8 µL min^−1^) for low shear or high flow rate (40 µL min^−1^) for physiological shear in BBB integrity studies.

The µE‐BBB assembled by adhering the subcomponents together as shown in **Figure** **2**, utilizing an arbor press to compress the device and increase the strength of the pressure sensitive adhesive. Polyethylene tubing (BTPE‐50, Instech) was inserted into the inlet and outlet ports of both lumen and basolateral chamber of the device and sealed with two‐part epoxide glue.

### 2.2 Cell Culture

Mouse brain endothelial cells (bEnd.3) (CRL‐2299, American Type Culture Collection) and mouse astrocyte cells (C8‐D1A) (CRL‐2541, American Type Culture Collection) were grown in Dulbecco's Modified Eagle Medium (DMEM) containing 10% fetal bovine serum and 1% Antibiotic Antimycotic Solution (Sigma‐Aldrich) vacuum filtered through a 0.22 µm polyvinylidene fluoride filter (complete growth media). The cells were grown in T‐75 culture flasks (Celltech) and grown to 70–90% confluency. The cells were passaged with 0.25%Trypsin/0.2%EDTA (Sigma‐Aldrich) and grown in a 37 °C incubator under 5% CO_2_.

The µE‐BBB was seeded with cells in two phases (Figure 2h,i). The initial seed of astrocytes was carried out on day 0, and the seed of endothelial cells was conducted on day 2. First, the device was sterilized by flowing 70% ethanol through both channels, followed by flowing phosphate‐buffered saline (PBS). The device was then filled with 50 µg mL^−1^ fibronectin and set to incubate for 1 h at 37 °C and subsequently filled with complete growth media and set to incubate for 2 h at 37 °C. Astrocytes were introduced into the basolateral chamber at a concentration of 6e^4^ cells cm^−2^. This was achieved by suspending the astrocytes to a concentration of 4e^5^ cells mL^−1^. After seeding, the µE‐BBB was placed upside down in the incubator. After 3 h, the device was perfused with complete growth media at a rate of 0.5 µL min^−1^. After 2 days, bEnd.3 cells were introduced to the luminal side at the same 6e^4^ cells cm^−2^ concentration, achieved by suspending the endothelial cells to a concentration of 1.2e^7^ cells mL^−1^. The device was then placed in the incubator for 3 h to allow for cell adhesion prior to perfusing the channel at a rate of 0.5 µL min^−1^ with complete growth media. Then, the cells within the µE‐BBB were allowed to grow for an additional 5 days, to reach full maturation as monitored by TEER trendline.^[^ ^40^ ^]^ Maturity was initially confirmed by imaging and subsequently determined by an asymptotic TEER trendline, over 170 Ω·cm^2^, comparable to similar models.^[^ ^40^, ^53^ ^]^

### 2.3 Immunocytochemistry

The µE‐BBB was washed with PBS and fixed with 4% paraformaldehyde on ice for 15 min. The adhesive maintaining the µE‐BBB was then weakened by submerging the device for 1 min. in isopropyl alcohol. The membrane was then excised from the µE‐BBB. Following blocking with 5% bovine serum albumin for 1 h at room temperature, bEnd.3 cells were incubated with Alexa 488 conjugated zonula occludens‐1 (ZO‐1) (Santa Cruz, sc‐33725 AF488) (1:100) or Alexa 488 conjugated claudin 5 (CLDN‐5) (352588, Invitrogen) (1:100) antibodies overnight at 4 °C. C8‐D1A cells were incubated with glial fibrillary acidic protein (GFAP) (13‐0300, Invitrogen) (1:100) overnight at 4 °C and with 488 goat anti‐rat secondary (A‐11006, Invitrogen) for 1 h at room temperature. Cells were then costained with DRAQ5 (62251, Invitrogen) (1:200) for 30 min. at room temperature. The membrane was mounted in fluoromount (00‐4958‐02, Invitrogen), and images were taken with the Leica SP5 Confocal Microscope. The bEnd.3 cells used for the evaluation of *S*‐Nitroso‐*N*‐acetyl‐DL‐penicillamine (SNAP) on ZO‐1 were stained similarly but were imaged with the Opera Phenix Microscope. Fiji software was used for processing the images.

### 2.4 TEER Validation

Daily TEER measurements were collected to monitor cell growth and the validation of TJ formation. This was done utilizing an EVOM2 epithelial voltohmmeter. Alligator clips were used to connect to the EVOM2 system via an EVOM2 Electrical Adapter. The initial resistance on day 0 was collected and represented the background resistance for the system. The TEER value, as represented by the equation below, was the product of the cross‐sectional growth areas within the system and the difference of the measured resistance from the initial day 0 resistance.

(2) $$\begin{array}{ccc} \left[\mathrm{TEER}\right]\Omega \cdot cm^{2} & = & \left[\mathrm{Raw}\;\mathrm{Resistance}-\mathrm{Raw}\;\mathrm{Resistanc}e_{\mathrm{Day}\;0}\right]\;\Omega \\ & & \times \left[\mathrm{CrossSectional}\;\mathrm{Area}\right]cm^{2} \end{array}$$

### 2.5 Fluorescein Isothiocyanate‐Dextran Permeability

The µE‐BBB permeability was further characterized by assessing the permeability of FITC‐dextran molecules across the barrier, from the lumen to the basolateral chamber. 10 kDa FITC‐dextran (HY‐128868, MCE) was dissolved to a concentration of 100 µg mL^−1^ in phenol red‐free DMEM. 400 µL of the solution was cycled through the lumen of the device at a rate of 40 µL min^−1^ via a peristaltic pump, with the basolateral chamber of the device filled with blank, phenol red‐free DMEM. After 1 h of perfusion, the circulating FITC‐dextran solution, the basolateral solution, and the stock FITC‐dextran (Ex. 470, Em. 514) solution were analyzed with a LightCycler 96 system (Roche). The concentrations of FITC‐dextran in the lumen and basolateral solutions were calculated with the following equation,

(3) $$\left[\mathrm{Lumen}\right]\;\frac{\mu g}{\mathrm{mL}}=\left(F_{L}-F_{B}\right)/\left(F_{S}-F_{B}\right)\;$$

(4) $$\left[\mathrm{Basolateral}\right]\;\frac{\mu g}{\mathrm{mL}}=\left(F_{\mathrm{BSL}}-F_{B}\right)/\left(F_{S}-F_{B}\right)$$

where *F*_S_ was the fluorescence of the stock solution, *F*_L_ was the fluorescence of the circulating lumen solution, and *F*_BSL_ was the fluorescence of the basolateral solution. The florescence of the blank media was denoted by *F*_B_. The permeability was measured on days 0, 2, 5, and 7.

### 2.6 Mouse Blood

Young male mouse blood (C57BL/6, 2–3 months) was purchased from BioIVT with sodium heparin as an anticoagulant. Old male mice (C57BL/6, 22–24 months) were purchased from the National Institute on Aging and all animal procedures were performed in accordance with the administrative panel of the Office of Laboratory Animal Care, UC Berkeley and the protocols were approved by the UC Berkeley Animal Care and Use Committee. Blood acquired from the old mice was collected via cardiac puncture into 2–3 units of heparin per milliliter. All blood was used within 24 h of collection.

### 2.7 Human Blood

Male human blood was purchased from BioIVT, with sodium heparin used as the anticoagulant. Young donors were 25–40 years of age and old donors were 65–80 years of age. Blood donors were de‐identified; however, samples were controlled for gender, BMI, race, and ethnicity. All blood was used within 24 h of collection. Donor information is provided in Table S1, Supporting Information.

### 2.8 Ex Vivo Effect of Nitric Oxide on Endothelial Cell ZO‐1 Expression

The bEnd.3 cells were seeded into a 96‐well, black half‐area plate (Corning 3882) at a concentration of 2e^4^ cells well^−1^. After 5 days of growth, NO‐donor, SNAP (Caymen Chemical, 80050), dissolved in phenol red‐free DMEM to a concentration of 1 mm, was incubated over the cells for 1 h. Following the SNAP treatment, the cells were fixed and imaged following the previously outlined protocol.

### 2.9 Ex Vivo Effect of Nitric Oxide on BBB

The bEnd.3 and C8‐D1A cells were seeded into the µE‐BBB using the protocol described above. Initial TEER measurements were collected to serve as a baseline pretreatment. After 7 days of growth, NO‐donor, SNAP (Caymen Chemical, 80050), dissolved in phenol red‐free DMEM to a concentration of 1 mm, was perfused through the µE‐BBB for 1 h. Following the SNAP treatment, post‐treatment TEER measurements were collected and FITC‐dextran permeability was measured.

### 2.10 Nitric Oxide Production Assay

Erythrocytes derived from young and old blood were isolated by Histopaque‐1077 (Sigma‐Aldrich) density separation. Isolated erythrocytes were resuspended to in vivo hematocrit levels with phenol red‐free DMEM, supplemented with 10 mm diaminofluorescein‐FM (DAF‐FM) (LSBio, LS‐H861). 400 µL of isolated erythrocytes were circulated through a modified µE‐BBB with a peristaltic pump at a rate of 40 µL min^−1^ for 1 h, inducing physiological shear stress (Figure 2j). The modified µE‐BBB did not feature a basolateral chamber, and only contained a shear inducing lumen layer. Low shear stress was induced by reducing the flow rate of the system to 8 µL min^−1^, achieving a shear stress of 0.4 Pa. Erythrocytes were pelleted at 600  *g* for 10 min and fluorescence measurements of the supernatant were collected (Ex. 470, Em. 514).

### 2.11 Free Hemoglobin Quantification Assay

Erythrocytes derived from young and old blood were isolated by Histopaque‐1077 (Sigma‐Aldrich) density separation. Isolated erythrocytes were resuspended to 0.5× in vivo hematocrit with phenol red‐free DMEM. 400 µL of isolated erythrocytes were circulated through a modified µE‐BBB with a peristaltic pump at a rate of 40 µL min^−1^ for 1 h, inducing physiological shear stress. The modified µE‐BBB did not feature a basolateral chamber, and only contained a shear inducing lumen layer (Figure 2j). Low shear stress was induced by reducing the flow rate of the system to 8 µL min^−1^, achieving a shear stress of 0.4 Pa. Erythrocytes were pelleted at 600  *g* for 10 min and the absorption at 520 nm of the supernatant was collected for pre‐ and post‐circulation under physiological shear stress. The relative change in absorption between these two values was quantified as Hb release under µE‐BBB physiological shear stress condition.

### 2.12 Erythrocyte Deformation Assessment

Erythrocytes derived from young and old blood were isolated by Histopaque‐1077 (Sigma‐Aldrich) density separation. Isolated erythrocytes were resuspended to 2.5% hematocrit with phenol red‐free DMEM. 400 µL of isolated erythrocytes were circulated through a modified µE‐BBB with a peristaltic pump at a rate of 40 µL min^−1^ for 1 h, inducing physiological shear stress. The modified µE‐BBB did not feature a basolateral chamber, and only contained a shear inducing lumen layer (**Figure** **2**j). Images were taken of the erythrocytes under 100× magnification with a Zeiss Axioskop 2 microscope fitted with a Hayer HY‐5299 camera.

**Figure 3:**
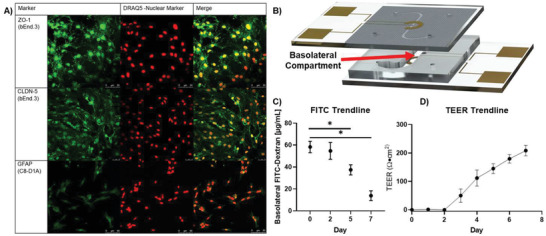
Validation of µE‐BBB. A) Excised membranes were stained for cell line specific markers. The bEnd.3 cells were stained for TJ markers, ZO‐1 and CLDN‐5, after 5 days of growth and the C8‐D1A cells were stained for astrocyte marker, GFAP, after 7 days of growth. DRAQ5 was used as a nuclear stain (Biological replicate: *n* = 1; technical replicates: *n* = 3). B) Schematic of the µE‐BBB lumen channel to the basolateral compartment where resistance of large molecule passage to the basolateral compartment was evaluated after 2, 5, and 7 days of growth. C8‐D1A cells were incorporated after day 0 measurements and bEnd.3 cells were incorporated after day 2 measurements. C) Large particle transport assays through µE‐BBB were conducted by passing 100 µg mL^−1^ of 10 kDa FITC‐dextran solution through the lumen compartment of the system for 1 h and quantifying the amount of FITC‐dextran that had accumulated in the basolateral compartment (*n* = 4; Mann–Whitney two‐tailed test; * signifies *p* < 0.05). Initially, there is little resistance to the diffusion of FITC‐dextran into the basolateral compartment, but as the device matures, the permeability of the system decreases. D) Utilizing the integrated electrodes of the system, TEER measurements were collected daily. During the first 2 days of culture, the astrocytes alone do not induce any change in resistance. However, the resistance begins to increase following incorporation of bEnd.3 cells after day 2 (*n* = 8). All data represented as mean ± SEM.

### 2.13 Ex Vivo Model of Mouse and Human Erythrocyte Shear on BBB

The bEnd.3 and C8‐D1A cells were seeded into the µE‐BBB using the previously described protocol and allowed to grow for 7 days. Human and mouse erythrocytes, derived from young and old donors, were isolated following the same Histopaque‐1077 (Sigma‐Aldrich) density separation protocol as previously described. Isolated erythrocytes were resuspended to 0.5× in vivo hematocrit levels with phenol red‐free DMEM, supplemented with 100 µg mL^−1^ FITC‐dextran. Initial TEER measurements were collected to serve as a baseline pretreatment. 400 µL of the isolated erythrocytes were perfused through the µE‐BBB for 1 h at 40 µL min^−1^ to induce physiological shear stress. Erythrocytes were perfused at 8 µL min^−1^ to induce low shear stress. Following erythrocyte perfusion, post‐exposure TEER measurements were collected and FITC‐dextran permeability was assessed. To isolate the erythrocyte from the FITC‐dextran solution, the cell suspension was centrifuged at 600  *g* for 10 min and the supernatant was collected.

### 2.14 Ex Vivo eNOS Inhibition

Human erythrocytes, derived from young and old donors, were isolated with Histopaque‐1077 (Sigma‐Aldrich) density separation. The isolated erythrocytes were incubated with 5 nm diphenyleneiodonium (DPI) in phenol red‐free DMEM for 10 min. The erythrocytes were subsequently washed and resuspended to 0.5× in vivo hematocrit levels with phenol red‐free DMEM.

### 2.15 Statistical Analysis

Any µE‐BBB devices exhibiting behavior of mechanical malfunction were excluded from consideration. Additionally, any µE‐BBB devices with baseline TEER values below the accepted plateau of ≈170 Ω·cm^2^ were removed as valid data points. The mean and standard error of the mean were presented in the results and figures and all statistical analysis were performed using Prism v8.4.3 For TEER data points; values represented were calculated by normalizing post‐treatment values to baseline measurements. No further data transformations were performed and no outliers were removed for any data set. The nonparametric Mann–Whitney two‐tailed test was utilized to determine the statistical significance of all data sets. Statistical significance was determined with *p* < 0.05 with *n* > 4 for each condition.

## 3 Results and Discussion

### 3.1 Optical Validation of Cell Growth in µE‐BBB Device

To verify BBB cell growth on the PC membrane, fluorescent images were collected of the bEnd.3 and the C8‐D1A) cell line. Immunocytochemistry staining for the cell‐fate markers confirmed integration of bEnd.3 and C8‐D1A cells into the BBB (**Figure** **3**a). The endothelial bEnd.3 cells express both TJ‐associated proteins, ZO‐1 and CLDN‐5^[^ ^54^ ^]^ after 5 days of growth within the *μ*E‐BBB system. Specifically, the well‐defined TJs are clearly identifiable with both markers. The C8‐D1A astrocyte cell line, cultured on the basolateral side of the membrane, are also identifiable by their expression of GFAP.^[^ ^55^ ^]^ Imaged after 7 days of culture, the C8‐D1A cells are morphologically distinct from the bEnd.3 cells grown on the opposite surface of the embedded membrane. Images were taken from single biological replicates of each condition with *n* = 3 technical replicates.

### 3.2 Validation of µE‐BBB Integrity

TJs play a pivotal role in establishing the barrier, however, following cell line introduction into the µE‐BBB, the TJ markers are not immediately robust. Several days of cell growth are required for TJs to properly develop within the BBB.^[^ ^56^, ^57^ ^]^ Accordingly, we evaluated the development of the barrier over the course of 7 days by monitoring two characteristics of a functional BBB, the decreased permeability and high electrical resistance. Permeability was monitored by quantifying the accumulation of 10 kDa FITC‐dextran^[^ ^40^, ^58^ ^]^ within the basolateral compartment of the µE‐BBB (Figure 3b), after circulating a 100 µg mL^−1^ solution of FITC‐dextran through the lumen of the system for 1 h (Figure 3c). As expected, there was no change in the permeability of FITC‐dextran by day 2. This is because astrocytes alone do not provide a functional barrier.^[^ ^31^ ^]^ However, after endothelial cells were incorporated into the device, a decrease in the basolateral accumulation of FITC‐dextran was observed on days 5 (*p* = 0.0286) and 7 (*p* = 0.0286). By day 7 the permeability of the device to FITC‐dextran was reduced by 24% relative to day 0. This decrease in permeability is associated with the establishment of a tighter barrier within the system.^[^ ^40^, ^53^, ^59^ ^]^

On‐chip TEER measurements of the µE‐BBB also demonstrated an increase (over 170 Ω·cm^2^) in the electrical resistance over time, after the incorporation of the endothelial cells (Figure 3d). This is in‐line with the progressive decrease in permeability and limited passage of ions across the integrated barrier after the endothelial cells are incorporated into the µE‐BBB.^[^ ^40^, ^59^ ^]^

### 3.3 The Effect of Nitric Oxide on ZO‐1 TJ Expression in Mouse BBB

**Figure 4:**
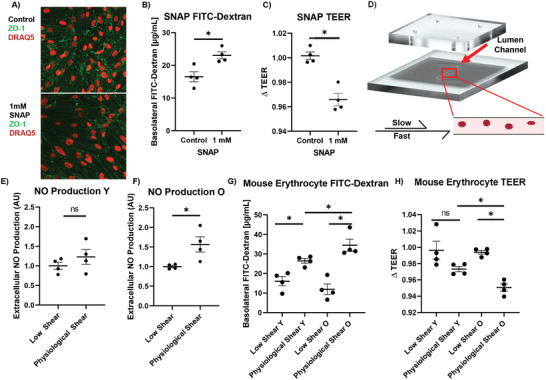
Evaluation of µE‐BBB response and mouse erythrocyte response to shear stress. A) ZO‐1 (green) expression was monitored after incubation with NO donor SNAP, showing a decrease in ZO‐1 expression in the treated condition. DRAQ5 (red) was used as a nuclear stain (Biological replicates: *n* > 3 and technical replicates: *n* = 9). B) To evaluate the effect of NO on the integrity of the barrier, 1 mm SNAP and FITC‐dextran were circulated through the µE‐BBB. The accumulated basolateral FITC‐dextran was quantified, indicating an increase in barrier permeability in the treated devices (*n* = 4). C) TEER evaluation of SNAP treated devices demonstrate a decrease in TEER values following the SNAP treatment, which further demonstrate damage to the µE‐BBB (*n* = 4). D) To evaluate the behavior of erythrocytes in response to shear stress, a modified µE‐BBB was utilized that only contains the shear inducing lumen channel. E) To quantify the production of NO by erythrocytes undergoing shear stress, isolated mouse erythrocytes, supplemented with NO probe DAF‐FM, were circulated through the modified µE‐BBB (*n* = 4). Under physiological shear stress conditions, there was no increase in the production of NO by young erythrocytes (*n* = 4). F) However, there was an increase in the production of NO from old mouse derived erythrocytes (*n* = 4). G) To evaluate the effect of erythrocytes on the permeability of the BBB, isolated erythrocytes from young or old mice, supplemented with FITC‐dextran, were circulated through the µE‐BBB. The accumulated basolateral FITC‐dextran demonstrates greater barrier permeability associated with old animal derived erythrocytes under physiological conditions (*n* = 4). H) TEER analysis mirrors this observation, demonstrating that the decrease in TEER was larger with old animal derived erythrocytes when compared to young animal derived erythrocytes, under physiological conditions (*n* = 4). Mann–Whitney two‐tailed test; ns, * signify *p* > 0.05 and *p* < 0.05, respectively. All data represented as mean ± SEM. (D) was adapted with permission from Biorender.com under a paid academic subscription.

NO is a molecule that increases BBB permeability.^[^ ^16^, ^17^, ^18^, ^19^ ^]^ To assess the effects of NO on TJ markers, immunocytochemistry for ZO‐1 was performed on endothelial cells that were exposed to the NO donor SNAP.^[^ ^60^ ^]^ Treatment with SNAP for 1 h resulted in a decrease in the fluorescence intensity of ZO‐1, indicative of decreased TJ expression (**Figure** **4**a).^[^ ^61^, ^62^ ^]^ Figure 4a represents images of *n* > 3 biological replicates and *n* = 9 technical replicates.

### 3.4 µE‐BBB Analysis of Nitric Oxide on the Permeability of Mouse BBB

To evaluate the effect of NO on the permeability of the µE‐BBB, the FITC‐dextran and TEER metrics employed in the studies in Figure 3c,d were used after the administration of SNAP for 1 h. The addition of SNAP resulted in an increase in the permeability of the µE‐BBB to FITC‐dextran (Figure 4b). Compared to the negative control, there was a 40% increase (*p* = 0.0286) in the accumulation of FITC‐dextran in the basolateral compartment. In addition, there was a 3.6% decrease in the TEER of the barrier (*p* = 0.0286) (Figure 4c). Both of these metrics are consistent with previous studies and signify damage to the BBB.^[^ ^40^, ^53^, ^63^, ^64^ ^]^

### 3.5 Production of Nitric Oxide from Young versus Old Mouse Erythrocytes

To confirm and extrapolate the capability of the µE‐BBB system to reflect the shear induced NO production, isolated erythrocytes were passed through the lumen of a modified µE‐BBB at different flow rates (40 or 8 µL min^−1^). Briefly, the modified µE‐BBB was designed without the basolateral compartment but was still capable of inducing physiological shear stress in the lumen compartment (Figure 4d). Erythrocytes were isolated from old and young animals, diluted to 0.5× in vivo hematocrit, mixed with the NO‐fluorescent probe DAF‐FM,^[^ ^65^ ^]^ and were circulated through the modified µE‐BBB at low (8 µL min^−1^) and physiological (40 µL min^−1^) shear rates for 1 h. The NO production was monitored. Physiological shear stress did not influence the NO production of young donor erythrocytes (Figure 4e), however, there was an increase in the old donor erythrocyte production of NO by 57% (*p* = 0.0286) (Figure 4f).

### 3.6 The Effect of Old and Young Mouse Erythrocytes on the µE‐BBB

To evaluate the role of mouse erythrocytes on BBB integrity, isolated mouse erythrocytes derived from young and old animals were circulated through the fully fitted µE‐BBB at low and physiological levels of shear stress. Erythrocytes resuspended to 0.5× in vivo hematocrit were mixed with 100 µg mL^−1^ 10 kDa FITC‐dextran and circulated through the µE‐BBB for 1 h, as described above. Subsequently, concentrations of basolateral FITC‐dextran were quantified and found to increase, suggesting a diminished µE‐BBB integrity that resulted from exposing circulating erythrocytes to physiological levels of shear stress (Figure 4g). Compared to low shear conditions, under physiological shear young animal‐derived erythrocytes showed an increase in FITC‐dextran permeability by 64.7% (*p* = 0.0286), while old animal‐derived erythrocytes showed an almost threefold greater increase in FITC‐dextran permeability, by 188.2% (*p* = 0.0286). The accumulated FITC‐dextran in the basolateral chamber of the µE‐BBB was 30.1% higher (*p* = 0.0286) after old animal‐derived erythrocytes were circulated under physiological conditions, when compared to young animal‐derived erythrocytes. Furthermore, TEER measurements confirmed the FITC‐dextran studies (Figure 4h). The decrease in TEER following exposure of young animal‐derived erythrocytes was not significant under physiological conditions, when compared to low shear conditions. However, the TEER decrease following the exposure of old animal‐derived erythrocytes was 4.4% larger (*p* = 0.0286) under physiological conditions, when compared to low shear conditions. The decrease in TEER under physiological conditions was 2.3% (*p* = 0.0286) larger with old animal‐derived erythrocytes when compared to young animal derived erythrocytes.

### 3.7 Quantification of Hemolysis from Isolated Human Erythrocytes from Different Age Groups

**Figure 5:**
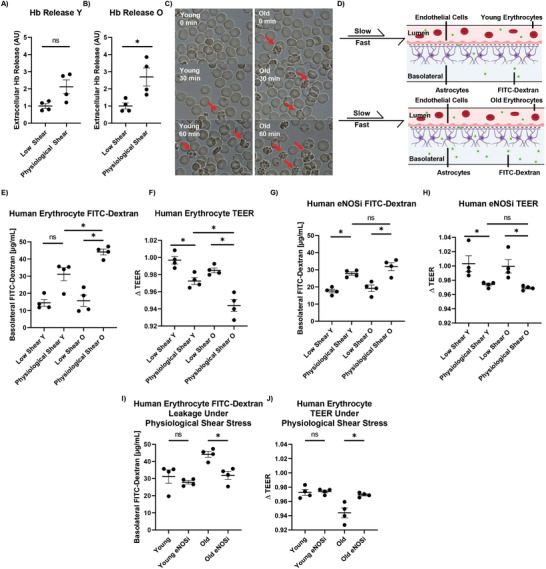
Effect of different age human erythrocytes on µE‐BBB. A) To quantify extracellular Hb, human erythrocytes were circulated through the modified µE‐BBB, and supernatant 520 nm absorbance was recorded to quantify free Hb. In relation to low shear conditions, young donor erythrocytes did not release more Hb than the low shear counterparts (*n* = 4), B) but old donor erythrocytes did (*n* = 4). C) To further evaluate erythrocyte response to shear stress, young and old erythrocytes were circulated through the modified µE‐BBB. After 1 h, there was a greater prevalence of apoptotic cells in the old erythrocyte population. D) To evaluate the effect of isolated human erythrocytes on the µE‐BBB, isolated young and old donor erythrocytes were circulated through the µE‐BBB supplemented with FITC‐dextran. The schematic shows that old and young erythrocytes experience either a slow flow rate (8 µL min^−1^) for low shear or high flow rate (40 µL min^−1^) for physiological shear for BBB integrity studies. Like the mouse erythrocyte studies, we expect a higher accumulation of basolateral FITC‐dextran in the old erythrocyte, physiological shear model. E) The accumulated basolateral FITC‐dextran demonstrates greater barrier permeability associated with old donor erythrocytes in relation to young donor erythrocytes under physiological conditions (*n* = 4). F) TEER analysis mirrors this observation, with a decrease in µE‐BBB electrical resistance associated with old‐derived erythrocytes when compared to young‐derived erythrocytes (*n* = 4). G) To evaluate the impact of erythrocyte produced NO on the µE‐BBB, eNOS inhibited (eNOSi) human erythrocytes were circulated through the µE‐BBB, and FITC‐dextran permeability shows no difference in the effect of young and old erythrocytes on the barrier (*n* = 4). H) TEER analysis of eNOS inhibited erythrocytes reaffirms the observation (*n* = 4). I) The permeability of the µE‐BBB to FITC‐dextran was lower in conditions where the old erythrocytes had been treated with the eNOS inhibitor, compared to the untreated sample (*n *= 4). J) Furthermore, there was a smaller decrease in TEER associated with this sample set (*n* = 4). Mann–Whitney two‐tailed test; ns, * signify *p* > 0.05 and *p* < 0.05, respectively. All data represented as mean ± SEM. (D) was adapted with permission from Biorender.com under a paid academic subscription. Components of Figure 5D created with BioRender.com.

As a surrogate marker of erythrocyte integrity, free Hb was quantified following exposure to low (8 µL min^−1^) and physiological (40 µL min^−1^) levels of shear stress using the modified µE‐BBB as depicted in Figure 4d. Isolated erythrocytes resuspended to 0.5× in vivo hematocrit, derived from young and old human donors, were circulated through the device and subjected to low and physiological shear stress for 1 h. The free supernatant Hb was then quantified with spectrophotometry absorbance (520 nm absorbance).^[^ ^66^ ^]^ Compared to low shear conditions, there was no increase in the amount of free Hb released by the young donor erythrocytes under physiological shear stress (**Figure** **5**a). However, there was an increase in old donor erythrocyte Hb release by 170% (*p* = 0.0286) after exposure to physiological shear stress (Figure 5b).

### 3.8 The Effect of Shear Stress on Young and Old Human Erythrocyte Morphology

We have shown that the µE‐BBB platform demonstrates the effects of old and young erythrocytes on the BBB in a mouse model. To expand this platform to the analysis of human erythrocytes, we first visualized the effect shear stress has on human erythrocyte morphology. Erythrocytes from young and old human donors were diluted to 2.5% hematocrit to allow for individual cell visualization and circulated through the modified µE‐BBB system for 1 h (Figure 4d). Still images were captured at 0, 30, and 60 min (Figure 5c). There were already more dead erythrocytes (diverging from its biconcave disk structure to an eryptosis structure^[^ ^67^, ^68^, ^69^ ^]^), in the old donor sample than the young donor sample before cell passage through the µE‐BBB (red arrows). Circulation of erythrocytes in the µE‐BBB increased the numbers of dead cells in both young and old samples; however, the rate of erythrocyte damage was greater in the old sample than in the young.

### 3.9 The Effect of Old and Young Human Erythrocytes on the µE‐BBB

Previous studies used mouse cell lines in vitro, to assess the response of human cells in drug screens^[^ ^70^, ^71^ ^]^ and specifically cocultured human and rodent cell lines, as a BBB mimic.^[^ ^72^, ^73^, ^74^, ^75^ ^]^ In our µE‐BBB, human erythrocytes were resuspended to 0.5× in vivo hematocrit and supplemented with 100 µg mL^−1^ 10 kDa FITC‐dextran. The suspension was circulated through the µE‐BBB for 1 h and the concentrations of basolateral FITC‐dextran were quantified (**Figure** **5d**). Results of this study show that when comparing old erythrocyte to young under physiological shear stress, there is an increase of 42% (*p* = 0.0355) in the µE‐BBB permeability (Figure 5e). Further corroborating this increase in permeability, a similar comparison of old to young erythrocyte circulation under physiological shear conditions was performed to assess TEER. The TEER measurements taken after circulating old human erythrocytes in µE‐BBB resulted in a 2.9% larger (*p* = 0.0078) decrease in resistance, when compared to young human erythrocytes (Figure 5f).

### 3.10 The Impact of eNOS Inhibition on Old and Young Human Erythrocytes

Old donor erythrocytes have been demonstrated to produce NO at a higher rate, under physiological shear stress, than their young counterparts. This is significant because NO causes a decrease of BBB integrity (Figure 4b,c). To confirm and extrapolate the damaging effect of NO on the BBB, the µE‐BBB permeability experiments were performed with erythrocytes that were exposed to the irreversible eNOS inhibitor (eNOSi), DPI. Erythrocytes from young and old human donors were isolated and resuspended to 0.5× in vivo hematocrit and supplemented with 100 µg mL^−1^ 10 kDa FITC‐dextran. The suspension was circulated through the µE‐BBB for 1 h and the concentrations of basolateral FITC‐dextran as well as TEER were quantified. In contrast to the diminished BBB integrity when untreated old cells were circulated in µE‐BBB under the physiologic shear stress (Figure 5e,f), there was no age‐specific difference in BBB permeabi flity when donor erythrocytes were pretreated with the eNOSi, DPI. (Figure 5g,h).

Exploring these phenomena in more detail, DPI did not change FITC‐dextran permeability with circulation of young erythrocytes in µE‐BBB. However, there was a 28% decrease in FITC‐dextran permeability with old erythrocytes, after treatment with DPI (Figure 5i). TEER analysis mirrored these results, with DPI having no effect of young donor erythrocytes, but resulting in a 3% smaller TEER decrease with old donor erythrocytes (Figure 5j).

## 4 Conclusion

Many factors contribute to BBB degradation, yet few are well understood in general and with respect to aging and disease. Here we present a novel organ‐on‐a chip technology which mimics physiologic circulatory changes and allows one to profile the effects of young and old environments (cells and molecules) on BBB integrity. Previous studies on heterochronic blood exchange^[^ ^1^ ^]^ and plasma manipulation^[^ ^76^ ^]^ have established a causal connection between the age‐associated hematological composition of an animal and brain health and function. Our study demonstrates that erythrocytes are playing an essential role through their effects on the BBB. These age‐specific effects are dependent on cell viability, deformability, and biochemistry, which were all quantitatively assayed within the µE‐BBB. Interestingly, dilution of old plasma with saline plus albumin improved brain health and function in mice^[^ ^76^, ^77^ ^]^ and attenuated progression of Alzheimer's disease in clinical trials.^[^ ^78^ ^]^ Albumin is known to change erythrocyte aggregation and sedimentation,^[^ ^79^ ^]^ thus it might preferentially sequester, neutralize, or buffer damaged old erythrocytes, thereby diminishing their negative effects on the BBB.

The reduction of TEER reported here is similar to that observed with hypoxia (9% reduction)^[^ ^80^ ^]^ and SARS‐CoV‐2 infection (5% reduction),^[^ ^63^ ^]^ although not reaching the magnitude of other pathologies like traumatic brain injury (46% reduction)^[^ ^81^ ^]^ or ethanol usage (20% reduction).^[^ ^64^ ^]^ Of note, the degradation of the barrier by the old erythrocytes in our system, is not akin to traumatic brain injury or hypoxia, in contrast, this is a cumulative impact of aged and the most prevalent in blood cells, which in µE‐BBB have detrimental effects on the barrier integrity in 1 h.

The age‐specific BBB changes are clearly due to multiple factors, and here we experimentally tested several of these. It has been hypothesized that enhanced erythrocyte eNOS activity is responsible for the degradation of the BBB. In this study, we demonstrated that NO affects TJ expression and induces BBB permeability. Furthermore, we established that old donor erythrocytes produce more NO under physiological shear stress than young. Additionally, the increase in BBB permeability by the old donor erythrocytes was reduced with eNOS inhibition. Additionally, it was hypothesized that increased fragility and higher rates of hemolysis of old erythrocytes have negative consequences for brain health,^[^ ^27^, ^28^ ^]^ which we confirmed and detailed for the effects on the BBB through the studies of cell morphology, cell death, and free Hb deposition.

Further research may identify erythrocytes as a target for therapy to promote healthy aging. Erythrocyte transfusions, a common FDA approved treatment for a variety of hematological conditions such as sickle cell disease or anemia, may help in improving BBB integrity. Furthermore, if erythrocyte rigidity is a cause of increased BBB permeability, therapeutics that increase erythrocyte membrane fluidity are expected to promote brain health in the old.

Technologically, this work innovates traditional models to study BBB ex vivo. The majority of systems utilize simplistic cell monolayers grown atop Transwell inserts.^[^ ^82^ ^]^ These platforms ignore the physiological shear stress present in vivo, which is important to consider, as it is a determinant of blood and endothelial cells properties and biochemistries.^[^ ^83^ ^]^ Additionally, our platform not only monitors the passage of various formulation across the BBB but it integrates highly sensitive electrodes across blood–brain interface to monitor TEER, as needed for accurate drug screens and for comparing candidate factors in their effects on the BBB. In commonly used TEER measurements systems, variations in the placement of the systems bulky, chopstick electrodes in‐between readings present a major source of error.^[^ ^84^ ^]^ Additionally, electrodes perpendicular to the growth area minimizes electrical noise by creating uniform flow of ions between the electrodes. The µE‐BBB with integrated perpendicular electrodes allows for reproducible real‐time monitoring of barrier integrity with much better accuracy. In addition, the design and material for the µE‐BBB allows rapid fabrication and are well scalable.

Beyond age associated impacts of specific blood components, this platform can serve to monitor the effects of other hematological pathologies on the integrity of the BBB. For example, the effect of sickle cell disease on BBB integrity could be studied as well as the impact of sickle cell disease treatments. Future work will also focus on incorporating additional cell lines, such as pericytes or neurons to provide a more functional neurovascular unit. Furthermore, human cell lines can be incorporated into the system to better simulate a humanized in vivo BBB environment for a closer clinical translation, including the data on the pharmacodynamic effects of biologics.^[^ ^57^, ^82^, ^85^, ^86^ ^]^

## Conflict of Interest

The authors declare no conflict of interest.

## Supporting information

Supporting Information

## Data Availability

The data that support the findings of this study are available from the corresponding author upon reasonable request.
