# Supplementary material for: Erythrocytes, a New Contributor to Age‐Associated Loss of Blood–Brain Barrier Integrity
Source: Adv Sci (Weinh). 2021 Aug 16;8(20):2101912. doi: 10.1002/advs.202101912 (PMC8529433; doi:10.1002/advs.202101912)
Supplement: Supplementary file 1 — Supporting Information [file ADVS-8-2101912-s001.pdf]

© 2021 Wiley-VCH GmbH

ADVANCED SCIENCE
Open Access

Supporting Information

for *Adv. Sci.*, DOI: 10.1002/advs.202101912

Erythrocytes, a new contributor to age-associated loss of blood-brain barrier integrity

Payam Amiri[1], *Jonalyn DeCastro*[1], *Joshua Littig*[1], *Hsiang-Wei Lu*[1], *Chao Liu*[2], *Irina Conboy*[2]*, *Kiana Aran*[1, 2]*

Supplementary Table 1: Human blood was collected from male donors and categorized into young (Y) (age-25-40) and old (O) (65-80) population groups. Blood donors were de-identified and all blood was used within 24 hours of collection.

Sample	Gender	Age	Ethnicity	BMI	Smoker	Medications:
Y1	Male	28	Black	21	Yes	None
Y2	Male	34	Black	32	Yes	None
Y3	Male	37	Black	21	No	None
Y4	Male	28	Caucasian	30	Yes	None
Y5	Male	39	Black	27	Yes	None
Y6	Male	40	Black	25	Yes	None
Y7	Male	29	Black	21	Yes	None
Y8	Male	30	Black	24	Yes	None
Y9	Male	37	Black	31	No	None
Y10	Male	36	Black	44	No	None
Y11	Male	26	Black	19	No	None
Y12	Male	28	Black	31	No	None
O1	Male	74	Hispanic	26	Yes	None
O2	Male	70	Hispanic	25	Yes	None
O3	Male	68	Caucasian	21	No	None
O4	Male	66	Black	24	Yes	High Blood Pressure Medication
O5	Male	80	Hispanic	28	Yes	None
O6	Male	76	Caucasian	24	Yes	None
O7	Male	66	Black	26	Yes	None
O8	Male	65	Black	26	Yes	None
O9	Male	67	Caucasian	22	Yes	None
O10	Male	66	Caucasian	29	Yes	None
O11	Male	71	Caucasian	29	No	None
